# An Evaluation of the Rate and Type of Orthodontic Tooth Movement When Injecting Platelet-Rich Plasma During Mini-Implant-Based Segmented en-Masse Retraction of Upper Anterior Teeth

**DOI:** 10.7759/cureus.62368

**Published:** 2024-06-14

**Authors:** Mohammad Wasim Al-Bozaie, Feras Baba, Mohammad Y Hajeer

**Affiliations:** 1 Department of Orthodontics, Faculty of Dentistry, University of Aleppo, Aleppo, SYR; 2 Department of Orthodontics, Faculty of Dentistry, University of Damascus, Damascus, SYR

**Keywords:** otm, acceleration of orthodontic tooth movement, type of orthodontic tooth movement, rate of orthodontic tooth movement, platelet-rich plasma, en-masse retraction

## Abstract

Introduction: The study aimed to determine the influence of palatal injection of platelet-rich plasma (PRP) on the rate and type of orthodontic tooth movement (OTM) during the en-masse retraction of upper anterior teeth.

Materials and methods: Two-arm parallel-group trial, in which 30 class II division 1 adult patients (7 males and 23 females) aged 16 to 27 years were recruited. The sample was randomly divided into two groups: the experimental group, in which PRP was injected in the palatal mucosa of the maxillary six anterior teeth immediately before starting the en-masse retraction of upper anterior teeth, whereas in the control group, traditional treatment was employed. Following the first premolar extraction, space closure was accomplished using frictionless mechanics for the en-masse retraction of upper anterior teeth. In both groups, a rigid segmented arch made of stainless steel with a diameter of 0.021 x 0.025 inch and an 8-mm power arm was used for the upper anterior teeth, and mini-implants were inserted between the upper second premolar and first molar at 8 mm apical from the archwire line. NiTi coil springs were used for retraction. Measurements were recorded at the onset of space closure (T0) and every 40 days till the middle of the en-masse retraction of upper anterior teeth (T1).

Results: Thirty patients completed the trial, and no patients were lost to follow-up in both groups. The OTM rate in the PRP group was similar to that of the control group (P = 0.596). The upper anterior teeth in the two groups were retracted mostly by controlled tipping and partially by translation. Statistically insignificant differences were observed between the two groups regarding the rest of the cephalometric variables. No serious harms were observed in either group.

Conclusions: PRP was ineffective in accelerating the OTM rate during the en-masse retraction of upper anterior teeth and it did not affect the type of tooth movement.

## Introduction

Adult patients with class II division 1 malocclusion may undergo camouflage treatment to correct their deformity by extracting upper premolars and retracting the upper anterior teeth posteriorly [1]. Retraction of upper anterior teeth can be done in one step (i.e., en-masse retraction of the six anterior teeth) or two steps (i.e., canine retraction followed by incisor retraction) [2]. Many studies have shown the superiority of the one-step technique compared to the two-step one [2,3]. Buccal fixed orthodontic appliances are the most common retraction method despite recent work employing lingual orthodontic devices [4]. Two biomechanical methods have been widely used for space closure in extraction instances during orthodontic treatment. One is the non-frictional or the segmented-arch technique [5,6], and the other is the sliding technique associated with friction during tooth movement [3,7]. Both methods have their advantages and disadvantages. With sliding mechanics, power arms fastened to a stainless steel archwire can be used to control the movement of the anterior teeth. However, the friction generated during retraction at the bracket-wire contact may slow down the movement of teeth in orthodontic treatment, lengthening the duration of treatment. Segmented arch technique has been devised to overcome the limitations of traditional techniques for space closure [8]. Because an archwire is split into three parts in this technique, friction is not created. This method has not been widely used.

Power arm and segmented arch mechanics have produced higher moment/force ratios without creating friction or vertical forces [8-11], allowing efficient and controlled tooth movement. In a three-dimensional finite element study [12], the researchers concluded that the power arm and segmented arch technique could provide a higher moment/force ratio sufficient for controlled anterior tooth movement without producing friction and vertical forces when applying retraction force parallel to the occlusal plane.

Orthodontists and patients are more aware of the recent advances in accelerated orthodontics [13]. Surgical procedures that accelerate orthodontic tooth movement (OTM), such as traditional corticotomy [14] and flapless piezocision [15], cause a planned injury to the cortical alveolar bone and lead to increased production of inflammatory cytokines that stimulate osteoclastic activity [16]. This ultimately leads to increased bone remodeling based on the regional acceleration phenomenon (RAP) [17]. This procedure creates a more elastic environment for the teeth to move in, thereby speeding up the OTM [18].

Platelet-rich plasma (PRP) is one of the biochemical methods to induce RAP, similar to surgical injury [19]. This causes the release of growth factors and cytokines, stimulates the activity of osteoblasts and osteoclasts, and interferes with the alveolar bone remodeling process. The submucosal injection of PRP is a technique described by Liou in 2016, claiming that the OTM might be accelerated by mimicking the effects of bone injury without requiring surgery and loss of alveolar bone. They argued that it could be applied for orthodontic tooth alignment and leveling in anterior crowding and space closure in en-masse anterior retraction or molar protraction [20].

Many animal studies have shown favorable results when PRP is used for accelerating OTM [21]. Several human studies have also discovered positive outcomes in favor of PRP [22-24]. Most of these studies included patients with isolated canine retraction. Conversely, several animal and human studies have concluded that PRP does not accelerate long-term [24-27]. Liou's research team assessed the en-masse retraction based on a case report in which he revealed that submucosal injection of PRP accelerated mandibular or maxillary alignment averaged 1.7 times [20]. In a recent randomized controlled trial, Chandak and Patil evaluated the influence of PRP on the OTM during the en-masse anterior retraction. According to their results, PRP did not influence the acceleration of the rate of OTM during the en-masse anterior retraction [28]. The study of Chandak and Patil injected the PRP from the buccal side, whereas the en-face surface of en-masse retraction movement is the palatal side, so they should have injected the blood concentrate palatally. The height of the power arm and the accurate positioning of used mini-screws were not provided in that study; therefore, it remains unclear whether the mechanics used or PRP had an impact on the type of OTM (i.e., bodily movement, controlled tipping movement, or a combination of them). The current trial's objectives were to evaluate PRP's effect on the rate and type of OTM during en-masse frictionless retraction of maxillary anterior teeth using a segmental arch technique. The null hypothesis was that injecting PRP did not affect the speed and type of OTM.

## Materials and methods

Study setup and design

This investigation used a two-arm parallel-group randomized controlled trial. From June 2019 to August 2022, this study was conducted at the Faculty of Dentistry, University of Aleppo, Aleppo, Syria. Before recruiting patients, the University of Aleppo Local Ethics Committee granted formal approval (Ref no. UADS-2420-24092019/SRC-1008). This trial was registered at the ClinicalTrials.gov database (Identifier: NCT06133361).

Estimating the sample size

The sample size was calculated using the G*power 3.1.9.4 program (Universität Düsseldorf, Düsseldorf, Germany) under the presumption of 80% power and a 5% allowable (α) error. The smallest difference requiring detection in en-masse retraction was assumed to be 0.5 mm/month with a standard deviation of 0.89 mm (from a previous study [28]). To account for sample attrition, four extra subjects were added to the required sample size of 26 participants for 30 patients.

Recruitment of participants and the criteria for eligibility

**Figure 1:**
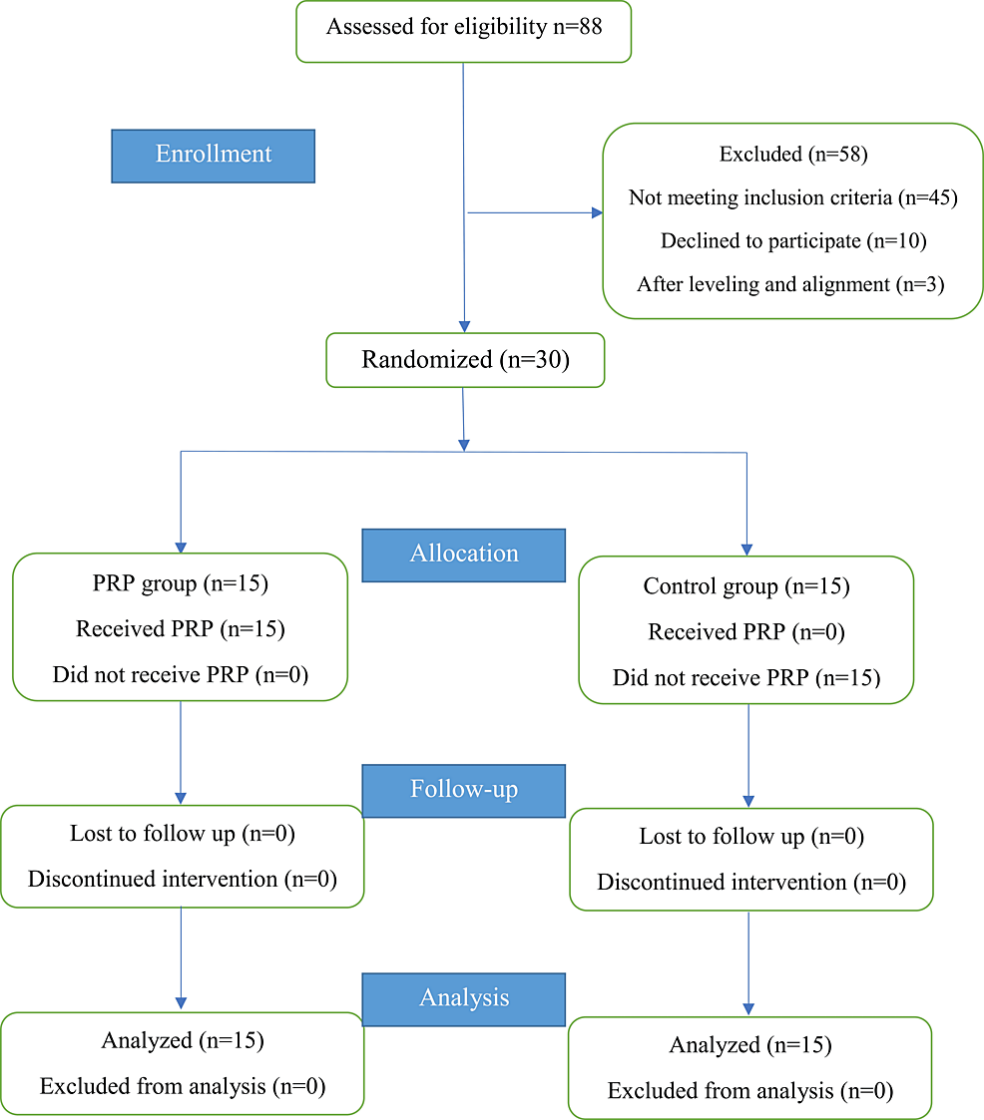
The consolidated standards of reporting trials (CONSORT) flow diagram for patient recruiting, follow-up, and entry into data analysis PRP: platelet-rich plasma

Recruitment was done by examining patients seeking orthodontic treatment at the Department of Orthodontics at the Faculty of Dentistry, University of Aleppo. Eighty-eight patients were examined, and the study project was explained to 43 patients who had met the inclusion criteria. Later, lateral cephalometric radiographs were performed for all patients to check their suitability for the study. Information sheets were given, and patients who agreed to participate in this study were asked to sign the related consent forms. Thirty-three agreed to participate. Depending on the calculated sample size, only 30 patients were selected randomly. The excluded patients were all treated at the Department of Orthodontics under the supervision of one of the co-authors. Patients' selection and follow-up are shown in Figure 1. The included patients were equally and randomly divided into group 1 (En-masse retraction with PRP injection, experimental group, n=15) and group 2 (En-masse retraction without PRP injection, control group, n=15).

All patients fulfilled the following inclusion criteria: 1) age range between 18 and 28 years, 2) class II division I malocclusion in which extraction of upper first premolars is indicated, 3) skeletal class II relationships (4° < ANB < 10°), 4) non-growing patients, 5) no maxillary constriction, 6) overjet >5 and <10 mm, 7) normal overbite of 0-50%, 8) normal anterior facial height, and 9) no systemic disease.

The exclusion criteria were: 1) patients who have undergone previous orthodontic treatment, 2) patients who need orthopedic surgery, 3) moderate to severe anterior crowding disharmony dento-maxillary (DDM) ≥ 3, 4) poor oral hygiene, 5) long-term use of medical drugs, especially NSAIDs, 6) Patients with habits, 7) patients with missing or extra teeth.

Randomization, blinding, and allocation concealment

One academic staff member who was not participating in the study performed simple randomization using computer-generated random numbers with a 1:1 allocation ratio. Sequentially numbered opaque sealed envelopes were used to hide the allocation order; they were unsealed only after the leveling and alignment process. Blinding was only used in the data analysis because it was difficult to blind both the principal investigator and the patients.

Orthodontic treatment in both PRP and control groups

In this study, two first upper premolars were extracted. Fixed metal orthodontic brackets (Majestic™, IOS, Boston, United States) with MBT prescription 0.022 were bonded in all patients. After the arches were leveled and aligned, a rigid sectional archwire (0.021 x 0.025 in stainless steel) was used for the anterior six teeth. To strengthen anchorage, microscrews (diameter 1.6 mm; length 10 mm; Genesis; Implant Genesis©, India) made of titanium alloy (self-drill type) were inserted at 8 mm from the archwire into the interradicular space between the maxillary first molar and second premolar after apical radiographs were taken.

**Figure 2:**
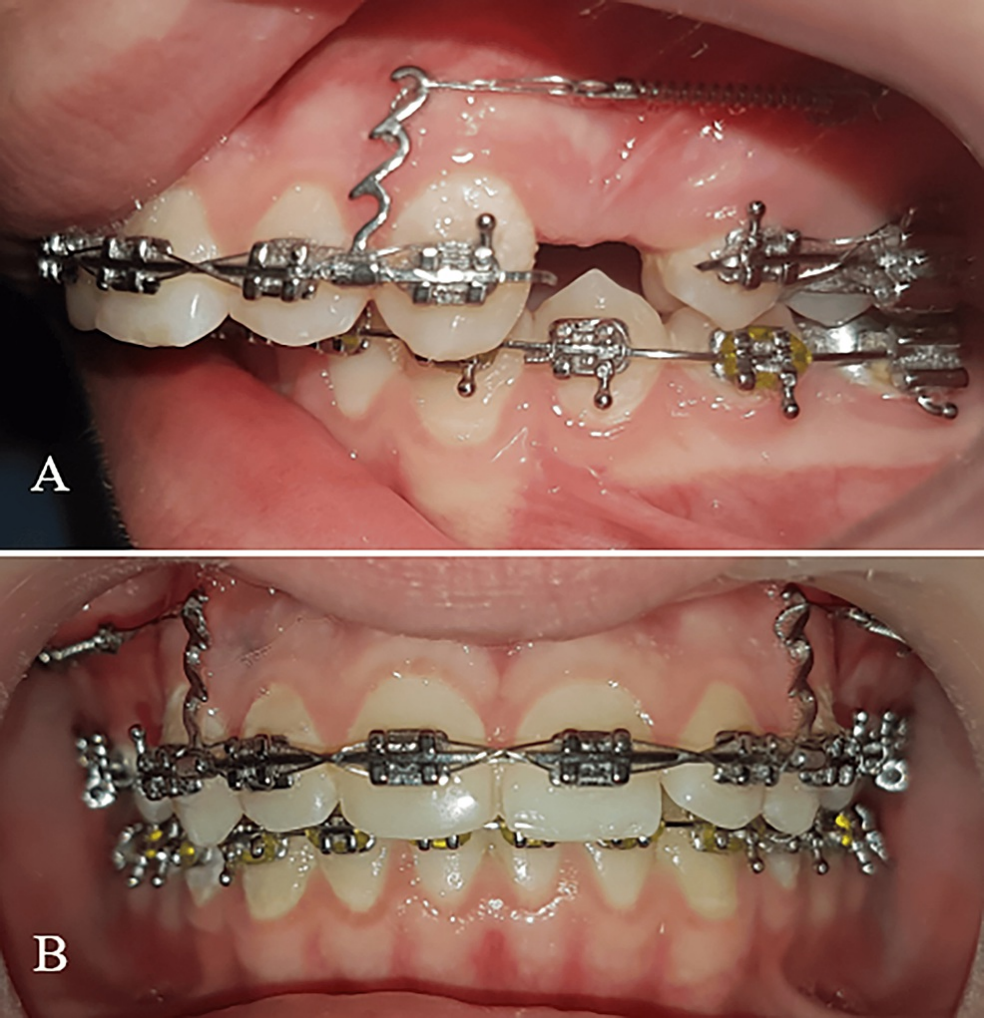
Frictionless mechanics (rigid segmented arch technique with an 8-mm power arm and miniscrew) for the en-masse retraction. A: lateral view. B: frontal view

Two crimpable hooks were used on both sides of the sectional wire 0.021x0.025, with a height of 8 mm between the lateral incisor and canine, to pass the force vector as close as possible to the center of resistance (Figure 2). The en-masse retraction began one week after premolar extraction, using calibrated nickel-titanium coil springs with 175 g force per side. The hooks, coil springs, and ligation wire are all adjusted to avoid irritation of soft tissues and ulcers.

PRP preparation and the site of injection in the PRP group

**Figure 3:**
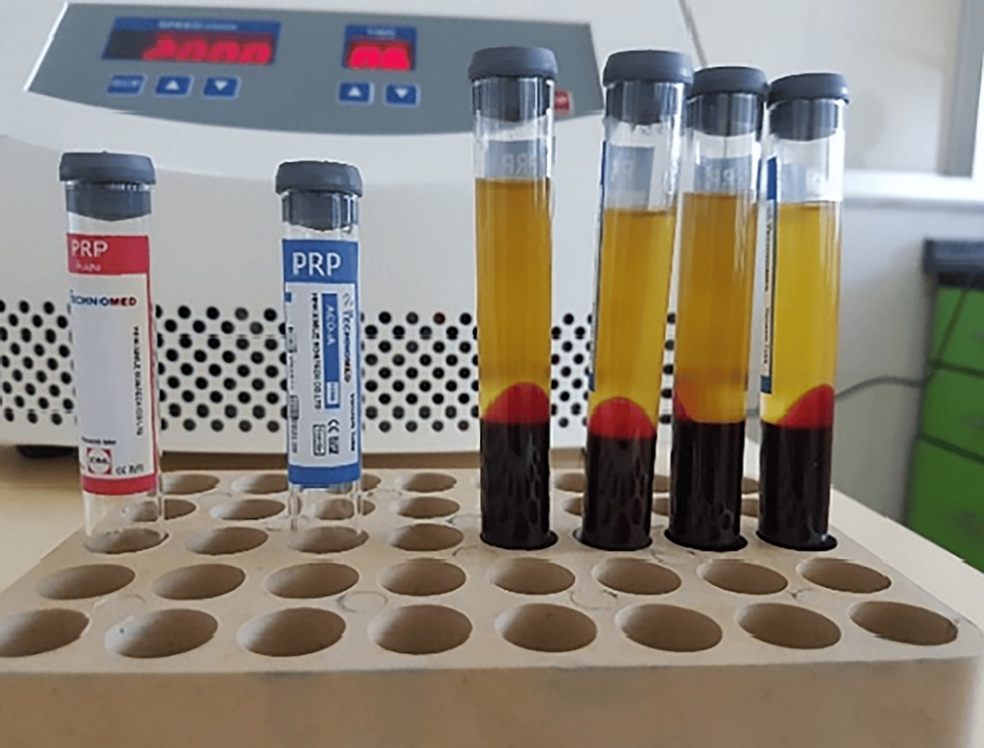
Separation of blood components into three different layers after first centrifugation PRP: platelet-rich plasma

**Figure 4:**
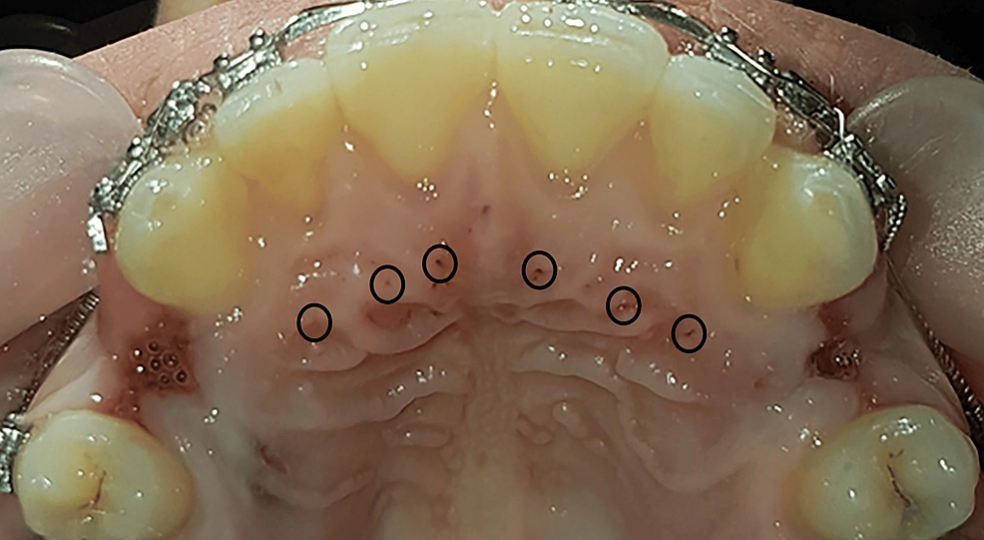
The injection of the platelet-rich plasma (PRP) into the palatal mucosa of maxillary six anterior teeth

About 48 ml of blood was directly collected by butterfly scalp vein from the patient to sterile tubes with acid citrate dextrose-A (ACD-A) as an anticoagulant. PRP was prepared as follows. Initially, the blood was centrifuged at 2000 rpm for six minutes (Figure 3). After the blood was separated, PRP and some platelet-poor plasma (PPP) were collected and mixed in a dry tube, and then a second centrifugation was done at 2700 rpm for three minutes. After the second centrifugation, the lower 1/3rd of the tube is PRP. About 4 ml of PRP was collected from the tube, and then the patient was injected with it. This double centrifugation protocol was mentioned in the review of Dhurat and Sukesh [29]. After regional anesthesia for pain control, 0.5 ml of PRP was slowly injected submucosally palatal to each tooth from the right to the left canine using a 1cc syringe (Figure 4). Paracetamol was described for the patient to control pain and ensure confirmation not to use ibuprofen or other NSAIDs [20]. The injection was applied one time. For ethical reasons, no placebo injections were used in the control group. Since the palatal injections usually are annoying.

Outcome assessment

To evaluate the rate of OTM, the distance between the lateral surface of the canine and the mesial surface of the second premolar was measured clinically by a digital caliper [14] at the beginning of space closure (T0) and every 40 days when the coil springs were controlled at 175 g. These measurements were considered an indicator of en-masse retraction speed, remembering that the second premolars were attached to the first molars in front of the mini-screws roots.

**Figure 5:**
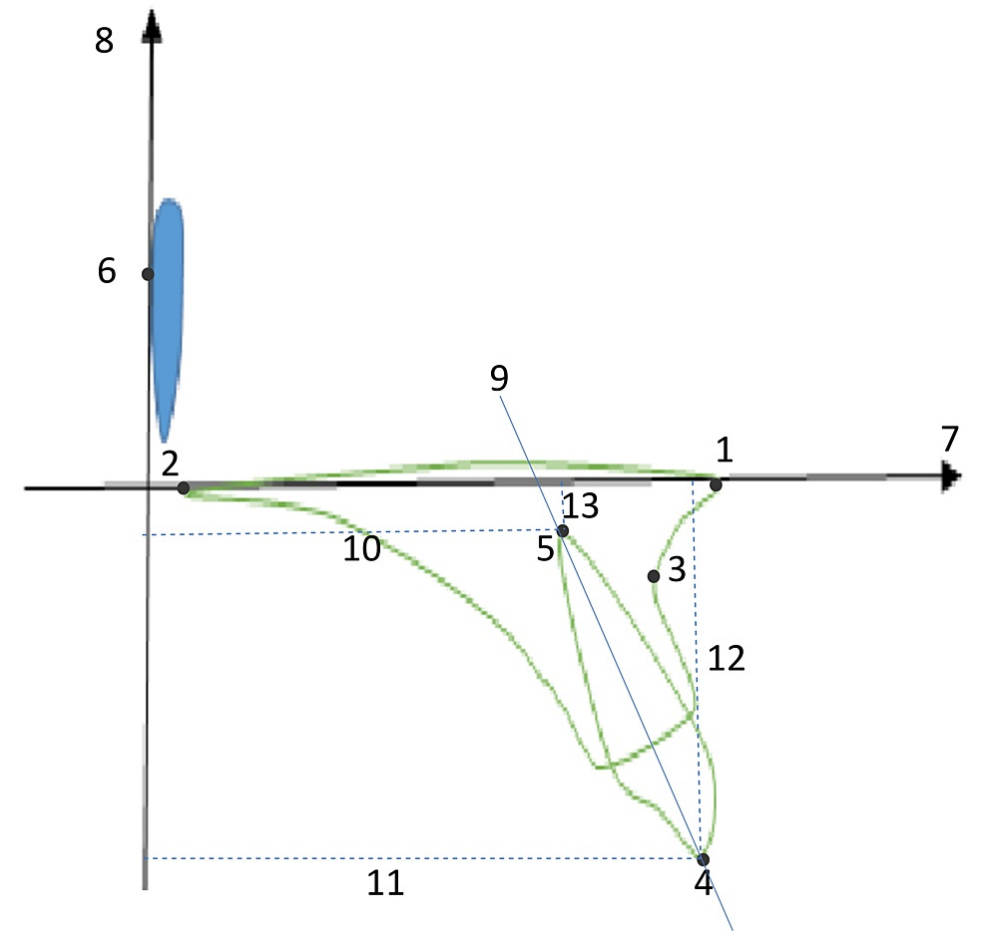
The cephalometric landmarks and planes utilized in this investigation Cephalometric landmarks: 1 - anterior nasal spine (ANS); 2 - posterior nasal spine (PNS); 3 - subspinale point (point A); 4- upper incisal edge (UIE); 5 - upper incisor apex (i.e., root apex; UIA); 6 - lateral point of the pterygoid fossa (lateral Pt point). Cephalometric planes: 7 - palatal plane (PP) or spinal plane (SPP; X-axis); 8 - line perpendicular to the SPP from lateral Pt point (Y-axis); 9 - upper incisor axis (UI); 10 - the antero-posterior movement of upper incisor apex (the distance from UIA to Y-axis); 11 - the antero-posterior movement of the upper incisal edge (the distance from UIE to Y-axis); 12 - vertical movement of upper incisal edge (the distance from UIE to X-axis); 13 - vertical movement of upper incisor apex (the distance from UIA to X-axis). All definitions are derived from Al-Sibaie and Hajeer [2] and Upadhyay et al. [30].

After reaching the middle of the overjet correction and about the middle of the extraction space closure, a lateral cephalometric radiograph named T1 was taken. Using a Pax-i3D cephalometer (Pax i3D, Vatech, Hwaseong-si, Korea), standardized lateral cephalometric radiographs were obtained at two assessment times: T0 (at the beginning of the en-masse retraction) and T1 (at the middle of en-masse retraction every patient was positioned in the cephalogram with their lips relaxed, their teeth in centric occlusion, the Frankfort plane parallel to the horizontal plane, and the X-ray path at a right angle to the sagittal plane. The definitions of the variables are provided in Table 1 according to Al-Sibaie and Hajeer [2] and Upadhyay et al. [30]. The cephalometric landmarks and planes are shown in Figure 5. All digitization, tracing, and analysis were performed by the principal researcher (MWA) using the FACAD program (FACAD, Ilexis AB, Linköping, Sweden). Two reference lines were established: the horizontal line (i.e., the X-axis) was drawn from ANS to PNS, which represented the palatal plane, whereas the vertical line (i.e., the Y-axis) was drawn perpendicular to the X-axis, passing through the lateral pterygoid point (Pt Point), as shown in Figure 5.

**Table 1:** Definition of angular and linear measurements used in the present study ^*^Definition according to Al-Sibaie and Hajeer [2]. ^**^Definition according to Upadhyay et al. [30]. NA: The line connecting the point Nasion (N) to point A (the deepest point on the anterior curvature of the alveolar bone at the upper incisor region, also called Subspinale point). NB: The line connecting the point Nasion (N) to point B (the deepest point on the anterior curvature of the alveolar bone at the lower incisor region).

Variables	Definition
SNA°	The angle between the anterior cranial base (i.e., the plane connecting the midpoint of the Sella Trurcia "S" to the most anterior point on the nasofrontal suture "N") and the NA plane.^*^
ANB°	The angle between the NA and NB planes.^*^
UI/SPP °	The angle between the long axis of the upper central incisor (UI) and the maxillary base plane.^*^
Overbite (mm)	The vertical overlap of the maxillary incisors over the mandibular incisors. This variable was measured relative to the incisal edges.^*^
UIE-v (mm)	The vertical distance between the upper incisal edge and the X reference plane (the line drawn from the anterior nasal spine (ANS) to the posterior nasal spine (PNS)).^**^
UIA-v (mm)	The vertical distance between the upper incisor apex (UIA) and the X reference plane.^**^
UIE-h (mm)	The horizontal distance between the upper incisal edge (UIE) and the Y reference plane (a line perpendicular to SPP at lateral PT point).^**^
UIA-h (mm)	The horizontal distance between the upper incisor apex (UIA) and the Y reference plane.^**^

The variables UIE-h and UIA-h represented the anteroposterior distance between the incisal edge and root apex, respectively, relative to the Y-axis. UIE-v and UIA-v represented the vertical distance between the incisal edge and root apex, respectively, relative to the X-axis, as shown in Figure 5. The type of tooth movement was determined by the ratio of root apex horizontal movement to incisal edge horizontal movement, as described by Upadhyay et al. in 2008 [30]. I/SPP angle and changes in point A (SNA and ANB) were measured. No changes to the study design were made after commencement, and the data analysis was planned to conform to the intention-to-treat principle.

Interim analyses

No interim analyses were applied in this trial.

Statistical analysis

Statistical analysis was performed using IBM SPSS Statistics for Windows, Version 25 (Released 2017; IBM Corp., Armonk, New York, United States). An independent-sample t-test was used to assess any significant inter-group differences. A significance level of 0.05 was chosen.

Assessment of the reproducibility of the measurements

The method's error was evaluated using Dahlberg’s formula on 18 randomly selected cephalograms (9 from the control group and 9 from the PRP group) [31]. These radiographs were re-measured by the same principal researcher (MWA) after a one-month interval. While intraclass correlation coefficients (ICCs) were utilized to assess intra-examiner reliability (i.e., random error), paired-sample t-tests were employed to identify systematic errors.

## Results

Baseline sample characteristics

In this trial, 30 patients were enrolled and randomly assigned to the PRP group or the control group. The results were based on an ITT analysis, not a per-protocol analysis. The overall sample age ranged from 18 to 26 years, with a mean age of 21.73 years (±1.79) for the PRP group and 22.46 years (±3.24) for the control group. All patients had skeletal class II with a mean ANB angle of 6.36° (±1.36°) in the control group and 6.16° (±1.32°) in the PRP group. Table 2 lists the baseline sample characteristics. The patients were committed, there was no chaos in the follow-up sessions, and there was no decrease in the sample size at the end of the study.

**Table 2:** Baseline sample characteristics at the beginning of the treatment PRP: platelet-rich plasma ^*^According to normal Bjork sum (396±6°) ^**^Overjet was divided into two categories: moderate (overjet greater than 5 mm and less than 8 mm) and severe (overjet ≥ 8 mm and less than 10 mm). ^***^Overbite was divided into two categories: normal (30% ≤ overbite ≤ 50%) and shallow (0% < overbite < 30%).

Group	PRP group	Control group
Number of patients	15	15
Sex distribution: male/female	4/11	2/13
Mean age (standard deviation)	21.73 (±1.79)	22.46 (±3.24)
Mean ANB (standard deviation)	6.16 (±1.32°)	6.36 (±1.36°)
Facial divergence angle (normal^*^)	395.56 (±4.06°)	397.06 (4.32°)
Overjet: moderate/severe^**^	6/9	7/8
Overbite: normal/shallow^***^	10/5	9/6

Reproducibility of the performed measurements

The error of the cephalometric measurements method based on Dahlberg’s calculations ranged from 0.19 to 0.36 mm for linear measurements and from 0.20 to 0.38 degrees for angular measurements and was considered low. No systematic error was detected using the paired t-test. The ICC confirmed the measuring procedure's high reliability, ranging from 0.802 to 0.996.

Main findings of the study

The mean retraction duration from T0 to T1 was about four months in both groups. The experimental group's mean amount of anterior retraction was 1.15±3.30mm, whereas the control group's was 3.09±0.99 mm (Table 3). The difference between the two groups was insignificant (P=0.596; Table 3). The calculated ratio representing the type of tooth movement was 0.23 (±1.36) and 0.42 (±0.87) in the PRP group and the control group, respectively (Table 3). This difference was statistically insignificant (Table 3).

**Table 3:** Descriptive statistics for the rate and type of OTM in both groups following half of the retraction stage with the p-values of significance testing* PRP: platelet-rich plasma; UIA: upper incisal apex; UIE: upper incisal edge; SD: standard deviation; PRPG: platelet-rich plasma group; CG: control group; CI: confidence interval ^*^Employing independent t-test. ^a^The horizontal movement was calculated as the difference in UIA-h and UIE-h between T0 and T1.

Variable	PRP group (n=15)		Control group (n=15)		PRPG-CG				
					Mean difference	95% CI lower bound	95% CI upper bound	p-value	
	Mean	SD	Mean	SD					
Amount of en-masse retraction	3.30 mm	1.15	3.09 mm	0.99	0.21	-1.013	0.593	0.596	
UIA horizontal movement^a^	0.83	1.62	1.02	1.96	-0.19	-0.787	1.167	0.487	
UIE horizontal movement	3.56	2.07	2.4	2.14	1.16	-1.967	3.13	0.537	
Apex M/edge M ratio	0.23	1.36	0.42	0.87	-0.19	-1.302	0.922	0.596	

The incisal edge moved vertically toward the X-axis by a mean of 0.27 mm. The root apex moved by a mean of 1.42 mm in the PRP group, meaning the upper incisors had an intrusion movement. In contrast, in the control group, the intrusion of the upper incisors was slightly greater than in the PRP group but was not statistically significant (the incisal edge and root apex moved in the vertical direction by 1.76 mm and 1.51 mm, respectively). Overbite decreased by 0.26 mm in the PRP group, whereas it decreased by 0.57 mm in the control group. Both angles, SNA and ANB, decreased by 0.47° and 0.48°, respectively, in the PRP group, whereas they decreased by 0.70° and 0.66°, respectively, in the control group. All cephalometric parameters for en-masse retraction were compared between the two groups, and the results showed statistically insignificant differences (Table 4).

**Table 4:** Descriptive statistics of the eight angular and linear cephalometric measurements with the p-values of significance testing* The definitions of these eight variables are given in Table 1. PRP: platelet-rich plasma; SD: standard deviation; T0: at the beginning of retraction; T1: at the middle of en-masse retraction; PRPG: platelet-rich plasma group; CG: control group; CI: confidence interval ^*^Employing independent t-test.

Variables	PRP group (n=15)			Control group (n=15)			Differences in the changes between the two groups			
	T0	T1	Mean difference T0-T1	T0	T1	Mean difference T0-T1	Mean difference	95% CI lower bound	95% CI upper bound	p-value
	Mean (SD)	Mean (SD)		Mean (SD)	Mean (SD)					
UIA-h (mm)	48.16 (3.16)	47.33 (3.11)	0.83	45.51 (4.66)	44.49 (4.80)	1.02	-0.19	-2.29	1.91	0.757
UIE-h (mm)	59.39 (3.55)	55.83 (4.15)	3.56	55.37 (5.91)	52.97 (6.61)	2.4	1.16	-0.97	2.13	0.184
UI/ SPP (°)	59.92 (7.64)	67.29 (8.12)	-7.37	63.87 (6.80)	67.39 (8.32)	-3.52	-3.85	-5.3	-2.4	0.295
UIE-v (mm)	26.46 )3.53)	26.19 (3.51)	0.27	26.82 (2.65)	25.06 (2.58)	1.76	-1.49	-2.06	-0.92	0.060
UIA-v (mm)	6.82 (2.08)	5.40 (2.34)	1.42	6.84 (2.47)	5.33 (3.11)	1.51	-0.09	0.75-	0.57	0.730
Overbite (mm)	3.08 (1.68)	2.82 (0.77)	0.26	3.43 (1.56)	2.86 (1.03)	0.57	-0.31	-1.09	0.47	0.240
SNA (°)	81.96 (2.98)	81.49 (2.83)	0.47	79.94 (4.39)	79.24 (3.88)	0.7	-0.23	-0.94	0.49	0.518
ANB (°)	6.16 (1.32)	5.68 (1.52)	0.48	6.24 (1.36)	5.58 (1.20)	0.66	-0.18	-0.81	0.45	0.569

## Discussion

This study was a single-blinded, two-arm, parallel-group randomized controlled clinical trial. According to our knowledge, it is the first trial in the literature to focus on the rate and type of OTM when retracting the upper anterior teeth using frictionless mechanics after injecting PRP. The choice of the en-masse retraction instead of the two-step retraction technique was dependent on several reports showing that the two-step technique requires more time to accomplish the retraction stage [2,3]. Frictionless mechanics with temporary anchorage devices (TADs) were employed in the current trial since this method eliminates problems caused by sliding mechanics, i.e., mainly the effect of friction [32-35]. In this study, mini-implants were used for anchorage for en-masse retraction. They are better than conventional anchorage systems, providing absolute anchorage and maximum retraction of the anterior teeth with good patient acceptance [36,37].

To maximize the bodily movement of the incisors during retraction, which was achieved in both groups, the 8-millimeter-height hooks allowed the force level to be adjusted. This allowed the line of action of force to pass through the center of resistance of the upper six anterior teeth as much as possible. Research has shown that the center of resistance of the upper six anterior teeth during en-masse retraction is around 7 mm apical to the interproximal bone level between the central incisors [38]. The study of Lee et al. in 2001 using three-dimensional finite element analysis pointed out that the center of resistance of the upper six anterior teeth was 6.76 mm apical to the cementoenamel junction of central incisors, and it moved apically (about 7.09 mm) when they applied corticotomy for the six anterior teeth [39]. In the study of Su et al. in 2014, it was suggested that the location of the center of resistance (CRe) of six maxillary anterior teeth is about 14 mm gingivally and 12 mm lingually to the incisal edge of the central incisor [40].

A rigid stainless steel wire with a diameter of 0.021 x 0.025-inch was used to connect the anterior teeth during retraction, and this is consistent with the recommendations made by Reimann et al. in 2007 for the centers of resistance of teeth to come close to each other, and for the teeth to look like a multi-rooted tooth which facilitates control of movement [41]. In a finite element study conducted in 2015, Ozaki et al. concluded that using a full-size archwire is advised to achieve controlled anterior tooth movement [12].

All the methods of chemically assisted acceleration of OTM increase the bone turnover rate and tooth movement rate. However, these chemical agents, i.e., hormones or vitamin D3, have many problems, such as systemic effects and short half-life, making them impractical in clinical orthodontics [17]. PRP contains cytokines (interleukins (ILs) and tumor necrosis factors (TNFs)), adhesive proteins, and leukocytes, considering that the OTM can be described as an inflammatory process; therefore, the presence of ILs and TNFs contributes to the acceleration of tooth movement [42]. In previously reported studies, PRP has been injected distal to the canines for canine retraction [22-24,26].

However, during the en-masse retraction of anterior teeth, the alveolar bone around each tooth must receive an equivalent quantity of osteoclastic stimulus for the teeth to move coherently. Keeping this in mind, in our study, PRP was injected into the palatal mucosa of all six anterior teeth by an insulin syringe. The blood sample was mixed with ACD-A as it is a good anticoagulant for PRP collection because it has the same effect regarding platelet count and the amount of released growth factor compared with sodium citrate [43]. The protocol of Dhurat and Sukesh [29] was followed in preparing the autologous PRP. An injectable PRP with a long-lasting effect is ideal for orthodontic applications [20,42]. PRP was made without combining thrombin or calcium dichloride as an activator to keep it liquid and injectable. Since a single injection of PRP has a clinical half-life of five to six months, it was injected once at the beginning of anterior retraction [20,26].

In this study, the overall rate of OTM (across the four-month observation period) was almost equal between the two groups, with an insignificant difference between them (P=0.596).

Chandak and Patil found that the rate of OTM (across a three-month observation period) was greater in the experimental group but also statistically insignificant. They suggested that it might be connected to a negative feedback mechanism in the growth factors' release, akin to the hormone-based negative feedback linked to elevated blood and/or tissue concentrations. [28]. El-Timamy et al. [24] noticed similar outcomes and concluded that normal growth factor production during OTM may have been impacted by elevating growth factor concentration in tissue that occurs incidentally after local PRP injection. Additionally, the literature reports on the systemic effects of submucosal PRP injection on liver function, which alter blood parameters such as calcium, alkaline phosphatase, gamma-glutamyl transferase, serum albumin, and serum total protein. This could slow the rate of OTM by causing an immune response against the therapy [22].

Both groups showed retraction by controlled tipping, which was close to a bodily movement, thus negating any error in the measurement of space closure by anterior retraction. Results showed a non-significant difference in the rate of en-masse retraction between the two groups (0.82 mm/month in the experimental group versus 0.77 mm/month in the control group). The same results were found in the Chandak and Patil Study [28]. Due to the lack of clinical studies on the effect of PRP on the rate of OTM, a comparison was made with the results of animal studies for both Güleç et al. and Rashid et al. Güleç et al. [19] found that PRP accelerated OTM by 1.4 to 1.7 times. Similarly, Rashid et al. [21] reported a higher value of acceleration (i.e., 2.13 times) compared to what was reported in the current trial. This may have resulted from the fact that their experiments were different in that they involved animals in more controlled settings and that there may have been a variation in the PRP composition. Point A retruded and SNA and ANB angles decreased. It could be because of partly bodily movement of upper incisors.

Limitations of the current study

The sample was primarily composed of female patients (80%); hence, it was impossible to investigate inter-gender differences. There was no way to blind the researcher or the patients, but this also couldn't be a source of bias because no patient-centered self-assessed outcomes, such as pain, discomfort, or swelling, were examined. Patients in the control group did not receive placebo injections, which could affect orthodontic movement due to the tissue damage caused by the injection, especially regarding palatal injections.

## Conclusions

Considering the limitations of this study, the following conclusions can be drawn: The rate of OTM was unaffected by the PRP. Frictionless en-masse retraction mechanics (a combination of a rigid anterior segmented arch and an 8-mm power arm) achieved controlled tipping closer to bodily movement in both groups. The anterior segment of the maxillary arch was retruded similarly between the two groups. Thus, the null hypothesis cannot be rejected in the current trial.

## References

[1] Khlef HN, Hajeer MY, Ajaj MA, Heshmeh O (2019). En-masse retraction of upper anterior teeth in adult patients with maxillary or bimaxillary dentoalveolar protrusion: a systematic review and meta-analysis. J Contemp Dent Pract.

[2] Al-Sibaie S, Hajeer MY (2014). Assessment of changes following en-masse retraction with mini-implants anchorage compared to two-step retraction with conventional anchorage in patients with class II division 1 malocclusion: a randomized controlled trial. Eur J Orthod.

[3] Khlef HN, Hajeer MY, Ajaj MA, Heshmeh O (2018). Evaluation of treatment outcomes of en masse retraction with temporary skeletal Anchorage devices in comparison with two-step retraction with conventional Anchorage in patients with dentoalveolar protrusion: a systematic review and meta-analysis. Contemp Clin Dent.

[4] Khattab TZ, Hajeer MY, Farah H (2022). Evaluation of the C-lingual retractor and the conventional lingual orthodontic brackets in terms of speech performance and oral discomfort: a randomized controlled trial. Cureus.

[5] Burstone CJ (1982). The segmented arch approach to space closure. Am J Orthod.

[6] Burstone CJ, Koenig HA (1976). Optimizing anterior and canine retraction. Am J Orthod.

[7] Bennett JC, McLaughlin RP (1990). Controlled space closure with a preadjusted appliance system. J Clin Orthod.

[8] Choy K, Pae EK, Kim KH, Park YC, Burstone CJ (2002). Controlled space closure with a statically determinate retraction system. Angle Orthod.

[9] El-Bialy TJJHS (2013). Segmented and sectional orthodontic technique: review and case report. J Health Spec.

[10] Park YC, Choy K, Lee JS, Kim TK (2000). Lever-arm mechanics in lingual orthodontics. J Clin Orthod.

[11] Sia S, Koga Y, Yoshida N (2007). Determining the center of resistance of maxillary anterior teeth subjected to retraction forces in sliding mechanics. An in vivo study. Angle Orthod.

[12] Ozaki H, Tominaga JY, Hamanaka R (2015). Biomechanical aspects of segmented arch mechanics combined with power arm for controlled anterior tooth movement: a three-dimensional finite element study. J Dent Biomech.

[13] Al-Ibrahim HM, Hajeer MY, Burhan AS, Sultan K, Ajaj MA, Mahaini L (2022). The efficacy of accelerating orthodontic tooth movement by combining self-ligating brackets with one or more acceleration methods: a systematic review. Cureus.

[14] Al-Naoum F, Hajeer MY, Al-Jundi A (2014). Does alveolar corticotomy accelerate orthodontic tooth movement when retracting upper canines? A split-mouth design randomized controlled trial. J Oral Maxillofac Surg.

[15] Al-Imam GM, Ajaj MA, Hajeer MY, Al-Mdalal Y, Almashaal E (2019). Evaluation of the effectiveness of piezocision-assisted flapless corticotomy in the retraction of four upper incisors: a randomized controlled clinical trial. Dent Med Probl.

[16] Mousa MR, Hajeer MY, Burhan AS, Heshmeh O (2023). Adult periodontal comparison after treatment of palatally impacted canines aligned by conventional or accelerated minimally-invasive corticotomy-assisted orthodontic traction: a randomized controlled trial. Int Orthod.

[17] Teixeira CC, Khoo E, Alikhani M (2017). Different methods of accelerating tooth movement. Clinical Guide to Accelerated Orthodontics.

[18] Frost HM (1983). The regional acceleratory phenomenon: a review. Henry Ford Hosp Med J.

[19] Güleç A, Bakkalbaşı BÇ, Cumbul A, Uslu Ü, Alev B, Yarat A (2017). Effects of local platelet-rich plasma injection on the rate of orthodontic tooth movement in a rat model: a histomorphometric study. Am J Orthod Dentofacial Orthop.

[20] Liou E (2016). The development of submucosal injection of platelet rich plasma for accelerating orthodontic tooth movement and preserving pressure side alveolar bone. APOS Trends in Orthod.

[21] Rashid A, ElSharaby FA, Nassef EM, Mehanni S, Mostafa YA (2017). Effect of platelet-rich plasma on orthodontic tooth movement in dogs. Orthod Craniofac Res.

[22] Ali Mahmood TM, Chawshli OF (2019). The effect of submucosal injection of plasma-rich platelets on blood inflammatory markers for patients with bimaxillary protrusion undergoing orthodontic treatment. Int J Inflam.

[23] Angel SL, Samrit VD, Kharbanda OP, Duggal R, Kumar V, Chauhan SS, Coshic P (2022). Effects of submucosally administered platelet-rich plasma on the rate of tooth movement. Angle Orthod.

[24] El-Timamy A, El Sharaby F, Eid F, El Dakroury A, Mostafa Y, Shaker O (2020). Effect of platelet-rich plasma on the rate of orthodontic tooth movement. Angle Orthod.

[25] Akbulut S, Yagci A, Yay AH, Yalcin B (2019). Experimental investigation of effects of platelet-rich plasma on early phases of orthodontic tooth movement. Am J Orthod Dentofacial Orthop.

[26] Liu L, Kuang Q, Zhou J, Long H (2021). Is platelet-rich plasma able to accelerate orthodontic tooth movement?. Evid Based Dent.

[27] Sufarnap E, Sofyanti E, Ilyas S (2018). The effect of platelet-rich plasma to orthodontic tooth movement. Proceedings of the International Dental Conference of Sumatera Utara 2017 (IDCSU 2017).

[28] Chandak S, Patil AS (2022). Effect of platelet-rich plasma on the rate of orthodontic tooth movement. Am J Orthod Dentofacial Orthop.

[29] Dhurat R, Sukesh M (2014). Principles and methods of preparation of platelet-rich plasma: a review and author's perspective. J Cutan Aesthet Surg.

[30] Upadhyay M, Yadav S, Patil S (2008). Mini-implant anchorage for en-masse retraction of maxillary anterior teeth: a clinical cephalometric study. Am J Orthod Dentofacial Orthop.

[31] Dahlberg G (1940). Statistical Methods for Medical and Biological Students. https://www.cabidigitallibrary.org/doi/full/10.5555/19402202514.

[32] AL-Suleiman M, Baba F, Suliman A, Swas O (2014). Clinical evaluation of upper incisors retraction frictionless system depending on mini-screw. J Dent Health Oral Disord Ther.

[33] Chung KR, Nelson G, Kim SH, Kook YA (2007). Severe bidentoalveolar protrusion treated with orthodontic microimplant-dependent en-masse retraction. Am J Orthod Dentofacial Orthop.

[34] Park YC, Chu JH, Choi YJ, Choi NC (2005). Extraction space closure with vacuum-formed splints and miniscrew anchorage. J Clin Orthod.

[35] Staggers JA, Germane N (1991). Clinical considerations in the use of retraction mechanics. J Clin Orthod.

[36] Barthélemi S, Desoutter A, Souaré F, Cuisinier F (2019). Effectiveness of anchorage with temporary anchorage devices during anterior maxillary tooth retraction: a randomized clinical trial. Korean J Orthod.

[37] Sandler J, Murray A, Thiruvenkatachari B, Gutierrez R, Speight P, O'Brien K (2014). Effectiveness of 3 methods of anchorage reinforcement for maximum anchorage in adolescents: a 3-arm multicenter randomized clinical trial. Am J Orthod Dentofacial Orthop.

[38] Vanden Bulcke MM, Burstone CJ, Sachdeva RC, Dermaut LR (1987). Location of the centers of resistance for anterior teeth during retraction using the laser reflection technique. Am J Orthod Dentofacial Orthop.

[39] Lee HK, Chung KR (2001). The vertical location of the center of resistance for maxillary six anterior teeth during retraction using three dimensional finite element analysis. Korean J Orthod.

[40] Su J, Liu J, Zhang D (2014). Finite-element investigation on center of resistance of maxillary anterior teeth (Article in Chinese). Sheng Wu Yi Xue Gong Cheng Xue Za Zhi.

[41] Reimann S, Keilig L, Jäger A, Bourauel C (2007). Biomechanical finite-element investigation of the position of the centre of resistance of the upper incisors. Eur J Orthod.

[42] Mangal UJB, Journal P (2017). Influence of platelet rich plasma on orthodontic tooth movement: a review. Biomed Pharmacol J.

[43] do Amaral RJ, da Silva NP, Haddad NF (2016). Platelet-rich plasma obtained with different anticoagulants and their effect on platelet numbers and mesenchymal stromal cells behavior in vitro. Stem Cells Int.

